# Higher level of physical activity reduces mental and neurological symptoms during and two years after COVID-19 infection in young women

**DOI:** 10.1038/s41598-024-57646-2

**Published:** 2024-03-22

**Authors:** Johanna Takács, Darina Deák, Akos Koller

**Affiliations:** 1 Department of Social Sciences, Faculty of Health Sciences, Semmelweis University, Budapest, Hungary; 2 Department of Morphology and Physiology, Faculty of Health Sciences, Semmelweis University, Budapest, Hungary; 3 Department of Translational Medicine, Faculty of Medicine, HUN-REN-SE Cerebrovascular and Neurocognitive Disease Research Group, Semmelweis University, Budapest, Hungary; 4 Research Center for Sport Physiology, Hungarian University of Sports Science, Budapest, Hungary; 5 Department of Physiology, New York Medical College, Valhalla, NY USA

**Keywords:** Auxin, Plant molecular biology, Screening, X-ray crystallography

## Abstract

Previous studies found that regular physical activity (PA) can lower the risk of SARS-CoV-2 (COVID-19) infection and post-COVID-19 condition (PCC), yet its specific effects in young women have not yet been investigated. Thus, we aimed to examine whether regular physical activity reduces the number of symptoms during and after COVID-19 infection among young women aged between 18 and 34 (N = 802), in which the confounding effect of other morbidities could be excluded. The average time since infection was 23.5 months. Participants were classified into low, moderate, and high PA categories based on the reported minutes per week of moderate and vigorous PA. Using the Post-COVID-19 Case Report Form, 50 different symptoms were assessed. Although regular PA did not decrease the prevalence of COVID-19 infection and PCC but significantly reduced the number of mental and neurological symptoms both in acute COVID-19 and PCC. Importantly, the high level of PA had a greater impact on health improvements. In addition, the rate of reinfection decreased with an increased level of PA. In conclusion, a higher level of regular PA can reduce the risk of reinfection and the number of mental and neurological symptoms in PCC underlying the importance of regular PA, even in this and likely other viral disease conditions.

## Introduction

The protective role of physical activity (PA) against a variety of respiratory infections has been established^1–3^. Previous studies highlighted the fact that regular PA, consistently meeting current guidelines on PA, improves immune function among many health-related benefits^4–6^; thus, PA lowers the risk for acute respiratory infections^7^ such as SARS-CoV-2 (COVID-19) infection. Indeed, a sedentary lifestyle is related to a higher risk of COVID-19 infection and increased severity of COVID-19 infection; regular PA can be a protective factor in reducing the risk of hospitalization and death^8–11^.

One-third of people who contracted SARS-CoV-2 experienced various long-lasting symptoms^12,13^, which may occur after asymptomatic or mild infection and persist for more than 4 weeks (post COVID-19 condition, PCC). The most commonly reported symptoms in PCC are fatigue, shortness of breath, changes in smell or taste, chest pain, difficulty sleeping, anxiety/depression, headache, and cognitive dysfunction^14^. Previous studies found that adherence to a healthy lifestyle (healthy body mass index, never smoking, high-quality diet, moderate alcohol intake, regular exercise, and adequate sleep) before infection was inversely associated with the risk of PCC in a ‘dose-dependent’ manner^15^. Being physically active can reduce post-infection fatigue and mental symptoms reducing the incidence and severity of PCC^16,17^.

Although symptoms of PCC are characterized by improvement and relapse phases, PA interventions normally improve the health-related quality of life reducing the number and severity of symptoms in PCC^18–21^. The Centers for Disease Control and Prevention (CDC) has identified risk factors for severe acute COVID-19, including advanced age, sex (male) and the presence of underlying comorbidities, such as diabetes, obesity and cardiovascular disease^22^, and these risk factors are also linked to physical inactivity. In general, there are differences in levels of PA between women and men; in most countries, women are less active than men^23^. Although males are more vulnerable to acute COVID-19, morbidity and mortality with acute SARS-CoV-2 have been higher in men, and PCC is more prevalent among women even after mild acute COVID-19 in a 4:1 female:male ratio^24–26^.

Finally, the prevalence of inactivity increases with increasing age; longitudinal studies showed that physical activity declines from adolescence to early adulthood. This change can continue throughout adulthood increasing health risks later in life^27–29^. The relationship between age and PCC has been controversial, previous studies found that age can be a significant predictor of PCC and a risk factor for PCC among women, but some studies did not find an association between age and PCC^26,30,31^.

Only a few studies examined young adults, particularly women, even though it has been found that persisting symptoms have a significant prevalence in young adults after a mild infection, especially among women^24–26^, who are less active than men^23^, resulting in negative impacts on general physical and mental functioning. We hypothesized that physically active women have fewer symptoms during and after COVID-19 infection compared to their inactive peers. Thus, the present study aimed to test the hypothesis that different levels of regular PA reduce various symptoms of acute COVID-19 and PCC among young women.

## Materials and methods

### Procedures and study sample

The data were collected between 20 July 2022 and 5 Oct 2023 and managed using REDCap electronic data capture tools hosted at Semmelweis University^32,33^. We were granted ethical approval by the Regional, Institutional Scientific and Research Ethics Committee, Semmelweis University (SE RKEB) (No. SE RKEB 118/2022). The study fully adheres to the ethical principles of the Declaration of Helsinki and all methods were carried out in accordance with relevant guidelines and regulations. Informed consent was obtained before completing the survey which includes information about the purpose of the study and the procedures. The participation was voluntary and the participants could withdraw from the study at any time. Each consent was implemented in REDCap using the survey functionality.

In the present study, we used a priori power analysis to calculate the sample size for a survey with a 0.95 confidence interval, 0.04 margin of error, and 0.95 confidence interval (Statistics Kingdom 2017; http://www.statskingdom.com). The calculated sample size was 601. During sampling, the sample size was doubled considering missing values, invalid cases and outliers, as well as the prevalence data of PCC and PA. Finally, based on the inclusion criteria, the required sample size was respected in the present study.

In sum, 1259 people filled out the questionnaire; 264 cases were invalid due to missing/incomplete data. For a homogenous study sample size in which comorbidities and gender effects are not present, males and older individuals were excluded from the final analysis. Based on the inclusion criteria, we excluded 193 cases who were older than 35 years (n = 55) and/or male (n = 147) and/or those who contracted SARS-CoV-2 less than 30 days before the data collection (n = 2). The study sample includes 802 women, aged between 18 and 34 years (M = 19.98, SD = 2.37).

### Measurements

Data on the infection and symptoms during (acute COVID-19) and after (post COVID-19 condition) COVID-19 infection were collected via an online survey using the WHO Post COVID Case Report Form (https://www.who.int/publications/i/item/global-covid-19-clinical-platform-case-report-form-(crf)-for-post-covid-conditions-(post-covid-19-crf-)). The Post COVID-19 Case Report Form includes 50 different symptoms which were put into ten major organ system categories: general, cardiovascular, neurological, mental, gastrointestinal, dermatological, musculoskeletal, pulmonary, ear-nose-throat and ophthalmological (ENT-O) and reproductive.

The Post COVID-19 Case Report Form also measured functioning as an indicator of the quality of life over the past seven days with 12 items on a 5-grade scale from ‘no difficulty’ to ‘extreme difficulty/cannot do’. In addition, these items were compared to the situation before COVID-19 on a 3-point scale (better, same, worse) to report changes in functioning. The total score of functioning and the changes in functioning were converted to an index between 0 and 100, taking into account the number of answered statements (functioning—0: no difficulty, 100: extreme difficulty/cannot do; changes in functioning—0: better, 50: same, 100: worse).

The level of PA was measured with the International Physical Activity Questionnaire short-form (IPAQ-SF)^34^, estimating regular physical activity levels. IPAQ-SF data were cleaned and processed according to the standardised scoring protocol. The reported minutes per week of moderate and vigorous activities were used to determine whether participants met the recommended physical activity levels of 150 min of at least moderate physical activity per week and to classify participants into low, moderate and high activity categories^35^.

### Data analysis

Descriptive statistics were reported in mean, standard deviation and relative frequencies. To examine group differences, one-way ANOVA was used with partial eta squared as effect size measurement. For testing association, Pearson’s chi-squared test was applied with Cramer’s V as the measure of association, and Pearson’s correlation coefficients were calculated. The level of significance was set at 0.05. Statistical analyses and visualization were conducted using IBM SPSS Statistics for Windows, Version 25.0 (IBM Corp. Released 2017, Armonk, NY, USA) and qgraph package (version 1.6.9.) in R^36^.

## Results

### Characteristics of COVID-19 infection

In the study sample, 55.1% of the young women (n = 442) contracted SARS-CoV-2; 84.1% (n = 370) of them had a confirmed COVID-19 infection (PCR, RAT, IgG/IgM) and 63.8% (n = 277) received a diagnosis of COVID-19 by a healthcare worker during the acute COVID-19. 9 cases had symptomatic acute COVID-19. Based on the classification of WHO, in most cases, the severity of the COVID-19 infection was mild (86.7%, n = 358) (moderate: 12.8%, n = 53, severe: 0.5%, n = 2). 92.3% of the participants (n = 740) received a COVID-19 vaccine (Pfizer-BioNTech > 80%), most of them in two (45.7%, n = 336) or three (50.7%, n = 373) doses. The reinfection rate was 38% (n = 167), mostly after vaccination (82%, n = 137). 26.3% (n = 114) of the participants reported persisting, still present or intermittent symptoms. The average time since infection was 23.46 months (SD = 10.35, min: 1.48 − max: 50.10).

### Characteristics of COVID-19 infection by level of PA

In sum, 42.9% (n = 344) of the women reported low, 34.8% (n = 279) moderate and 22.3% (n = 179) high PA. The prevalence of COVID-19 infection and the PA categories showed a significant moderate association (χ^2^(2,N = 802) = 6.433, *p* = 0.040, V = 0.09). A higher rate of COVID-19 infection was found in the moderate PA group (60.9%, n = 170) compared to the low (53.2%, n = 183) and high PA (49.7%, n = 89) groups.

The severity of the COVID-19 infection was mild in most cases regardless of the level of PA. However, the two severe cases showed low PA, and the rate of moderate severity was higher in the high PA group (20%, n = 16) than in the low PA (10.9%, n = 19) and moderate PA (11.3%, n = 18) groups.

Reinfection and PA did not show a statistically significant association (χ^2^(2,N = 440) = 3.978, *p* = 0.137, V = 0.10). Based on the frequency data, with a moderate effect, the rate of reinfection decreased with the increase in the level of PA (low PA: 43.3%, n = 78; moderate PA: 35.3%, n = 60; high PA: 32.2% n = 29). There was no statistically significant association between PCC and PA (χ^2^(2,N = 433) = 0.581, *p* = 0.748, V = 0.04). The prevalence of PCC was one-quarter regardless of the level of PA (low PA: 26.1%, n = 46; moderate PA: 28%, n = 47; high PA: 23.6 (n = 21).

### Symptoms in acute COVID-19 by level of PA

During acute COVID-19, an average of 13.91 ± 10.41 (min = 1, max = 49) symptoms were reported; 10% of the cases (n = 44) were asymptomatic. The number of symptoms decreased with the level of PA (F(2,380) = 5.024, *p* = 0.007, η^2^_p_ = 0.03). The number of symptoms was the lowest in the high PA group (M = 11.64, SD = 10.79), while it was the highest in the low PA group (M = 15.88, SD = 10.79) and the number of symptoms was 13.12 (SD = 9.93) in the moderate PA group.

**Figure 1:**
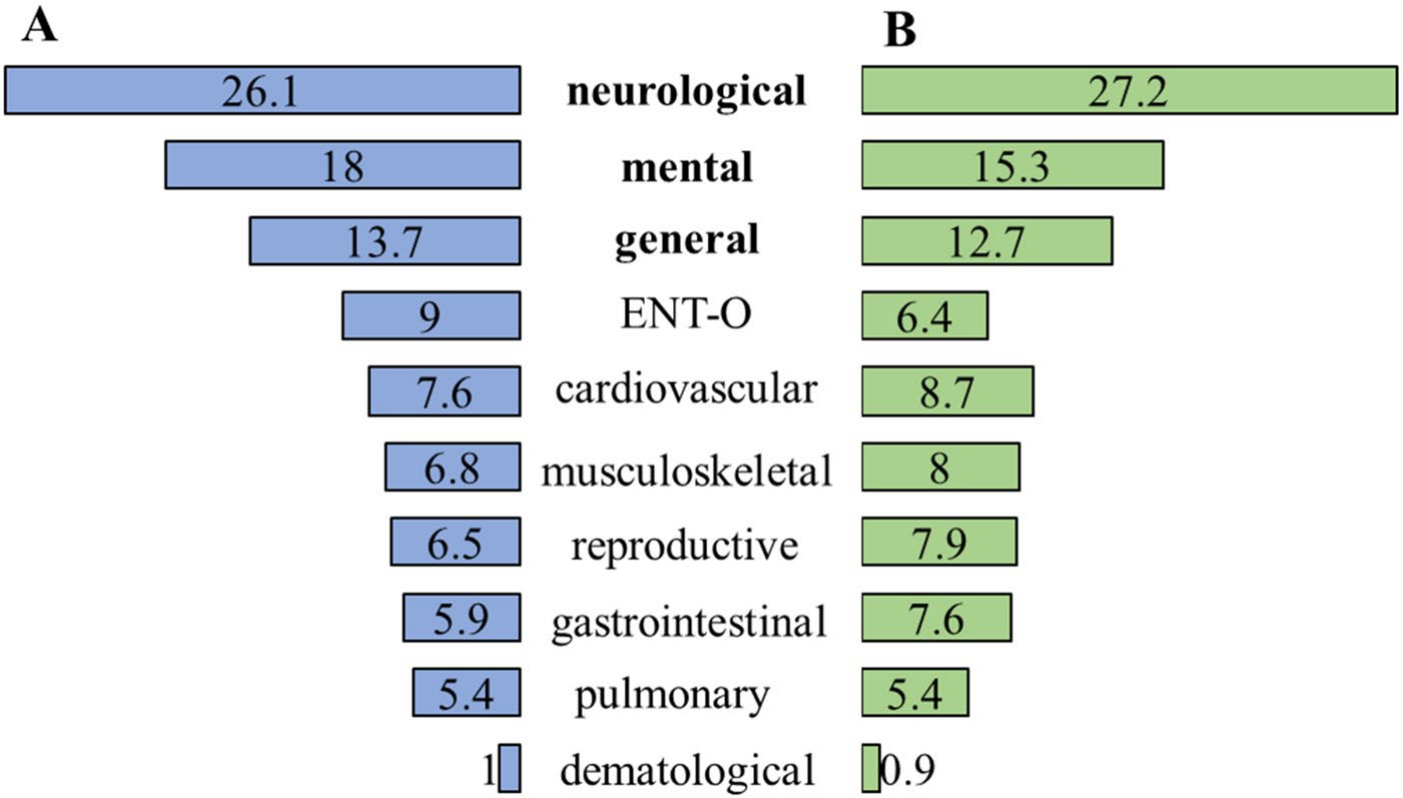
Relative frequency distribution (%) of various symptoms of organ systems in acute COVID-19 (**A**) and in post COVID-19 condition (**B**), show that most symptoms were neurological, mental, and general symptoms, in acute COVID-19 and PCC. *Notes* The values are shown in percentage. ENT-O: ear-nose-throat and ophthalmological symptoms.

Most of the symptoms were neurological, mental and general, regardless of the level of PA (Fig. 1A). At the same time, the symptoms of these organ systems more frequently occurred in the low and moderate PA groups compared to the high PA group (Table 1).

**Table 1:** Frequency of symptoms of organ systems in acute COVID-19 in the total sample and the PA categories. Notes ENT-O: ear-nose-throat and ophthalmological symptoms.

	Total sample	Physical activity categories			χ^2^	*p*	V
		Low	Moderate	High			
	%(n)	%(n)	%(n)	%(n)			
Neurological	84.6 (324)	**90.0 (135)**	**84.2 (133)**	**74.7 (56)**	**9.057**	**0.011**	**0.15**
Mental	81.7 (313)	**85.3 (128)**	**84.8 (134)**	**68.1 (65)**	**11.773**	**0.003**	**0.18**
General	78.1 (299)	**85.3 (128)**	**75.9 (120)**	**68.0 (51)**	**9.479**	**0.009**	**0.16**
Reproductive	61.0 (233)	66.0 (99)	59.2 (93)	54.7 (41)	3.046	0.218	0.09
Cardiovascular	59.3 (227)	62.7 (94)	57.0 (90)	57.3 (43)	1.182	0.554	0.06
Musculoskeletal	54.3 (208)	60.0 (90)	51.9 (82)	48.0 (36)	3.531	0.171	0.10
ENT-O	53.5 (205)	58.0 (87)	46.2 (73)	60.0 (45)	5.877	0.053	0.12
Gastrointestinal	49.6 (190)	51.3 (77)	50.0 (79)	45.3 (34)	0.737	0.692	0.04
Pulmonary	49.6 (190)	57.3 (86)	44.3 (70)	45.3 (34)	5.907	0.052	0.12
Dermatological	13.6 (52)	18.0 (27)	10.8 (17)	10.7 (8)	4.111	0.128	0.10

Significant values are in [bold].

**Figure 2:**
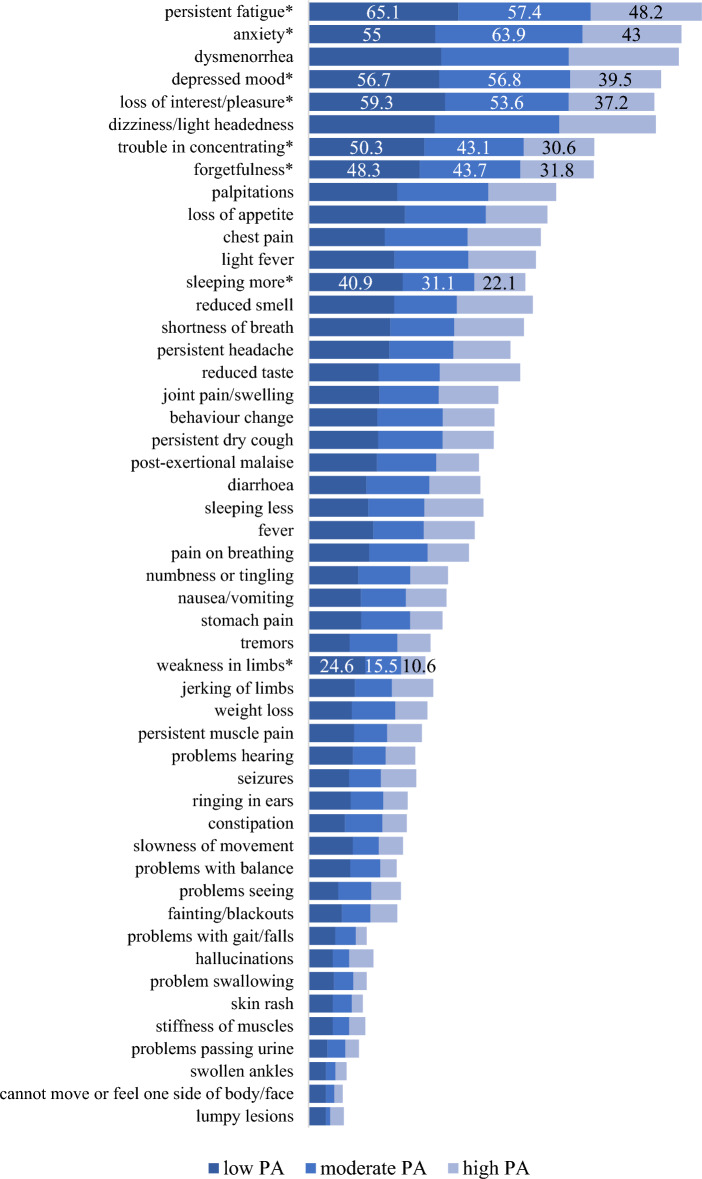
Frequency of symptoms in acute COVID-19 by the level of PA (*: significant association between the symptom and the low, moderate and high PA groups; PA: physical activity; the values are shown in percentage).

In sum, the most frequently reported symptoms were fatigue (58.7%), anxiety (56.1%), dysmenorrhea (54.7%), depressed mood (53.3%), loss of interest/pleasure (52.6%), dizziness/light headedness (51.9%), trouble in concentrating (43.5%) and forgetfulness (43.2%). In the case of these symptoms, the frequency (%) was higher in the low and moderate PA groups compared to the high PA group (Fig. 2). For data on all the symptoms in the sample and the PA categories, see Supplementary File 1.

### Symptoms in post COVID-19 condition by level of PA

In post COVID-19 condition, an average of 11.89 ± 8.60 (min = 1, max = 38) symptoms were experienced. The number of symptoms decreased in the high PA group (M = 8.12, SD = 7.66) compared to the low (M = 11.02, SD = 8.45) and moderate (M = 14.07, SD = 8.62) PA groups, after adjusting time since infection (F(2,102) = 3.474, *p* = 0.035, η^2^_p_ = 0.06). The majority of the symptoms were neurological, mental and general, regardless of the level of PA (Fig. 1B). At the same time, neurological and mental symptoms more frequently occurred in the low and moderate PA groups compared to the high PA group. In addition, the frequency of cardiovascular symptoms was higher in the moderate PA group than in the low and high PA groups (Table 2).

**Table 2:** Frequency of symptoms of organ systems in post COVID-19 condition in the total sample and the PA categories. Notes ENT-O: ear-nose-throat and ophthalmological symptoms.

	Total sample	Physical activity categories			χ^2^	*p*	V
		Low	Moderate	High			
	%(n)	%(n)	%(n)	%(n)			
Neurological	86.7 (91)	**90.5 (38)**	**93.5 (43)**	**58.8 (10)**	**13.779**	**0.001**	**0.36**
Mental	78.1 (82)	**81.0 (34)**	**84.8 (39)**	**52.9 (9)**	**7.691**	**0.021**	**0.18**
General	77.1 (81)	76**.**2 (32)	80.4 (37)	70.6 (12)	0.719	0.698	0.08
Cardiovascular	63.8 (67)	**57.1 (24)**	**78.3 (36)**	**41.2 (7)**	**8.739**	**0.013**	**0.29**
Reproductive	60.0 (63)	59.5 (25)	63.0 (29)	52.9 (9)	0.534	0.766	0.07
Musculoskeletal	52.4 (55)	50.0 (21)	58.7 (27)	41.2 (7)	1.686	0.430	0.13
Gastrointestinal	51.4 (54)	54.8 (23)	54.3 (25)	35.3 (6)	2.115	0.347	0.01
Pulmonary	42.9 (45)	35.7 (15)	52.2 (24)	35.3 (6)	2.902	0.234	0.02
ENT-O	39.0 (41)	31.0 (13)	45.7 (21)	41.2 (7)	2.032	0.362	0.14
Dermatological	9.5 (10)	9.5 (4)	10.9 (5)	5.9 (1)	–*	–	–

*The expected count of cells was less than 5.

Significant values are in [bold].

**Figure 3:**
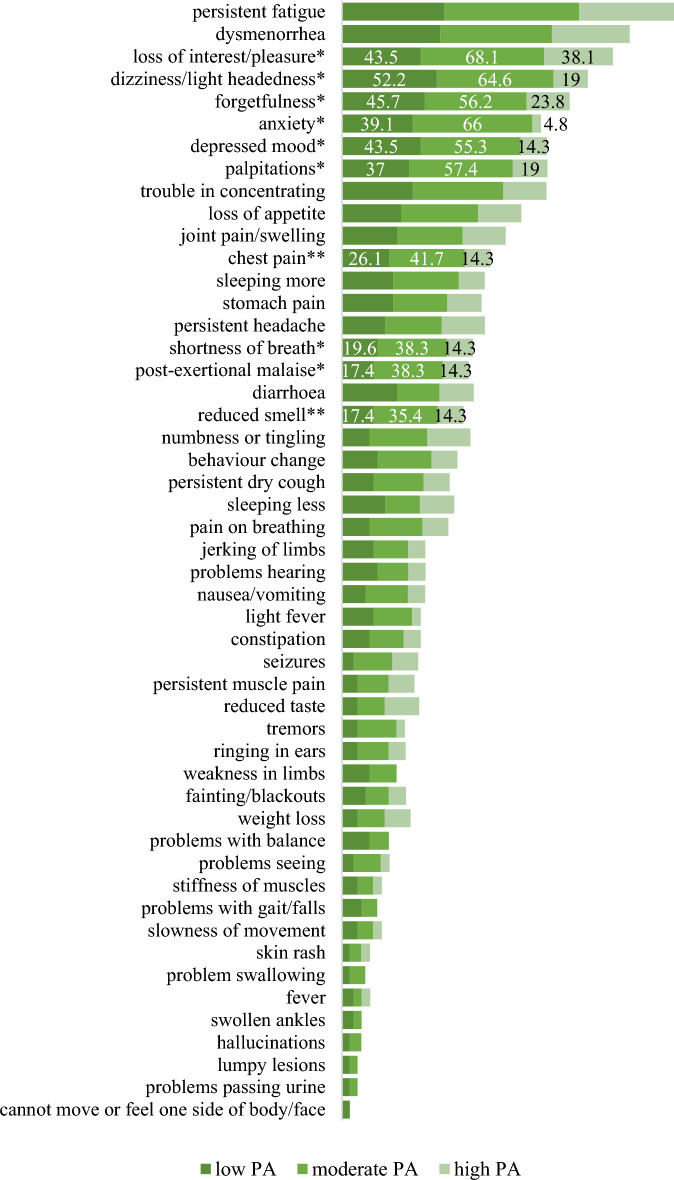
Frequency of symptoms in post COVID-19 condition by the level of PA (*: significant association between the symptom and the low, moderate, and high PA groups; PA: physical activity, the values are shown in percentage).

In sum, the most frequently reported symptoms were fatigue (63.2%), dysmenorrhea (55.3%), loss of interest/pleasure (52.6%), dizziness/light headedness (51.3%), forgetfulness (46.1%), anxiety (43.9%), depressed mood (43%), palpitations (42.1%) and trouble in concentrating (40.9%). In the case of depressed mood, forgetfulness and dizziness/light headedness, a higher frequency was shown in the low and moderate PA groups compared to the high PA group, while in the case of the following symptoms, it was the moderate PA group which showed a higher frequency compared to the low and high PA groups: loss of interest/pleasure, anxiety and cardiovascular symptoms such as palpitations, chest pain, post-exercise malaise and reduced smell as well as shortness of breath (Fig. 3). For data on all the symptoms in the sample and the PA categories, see Supplementary File 2.

**Figure 4:**
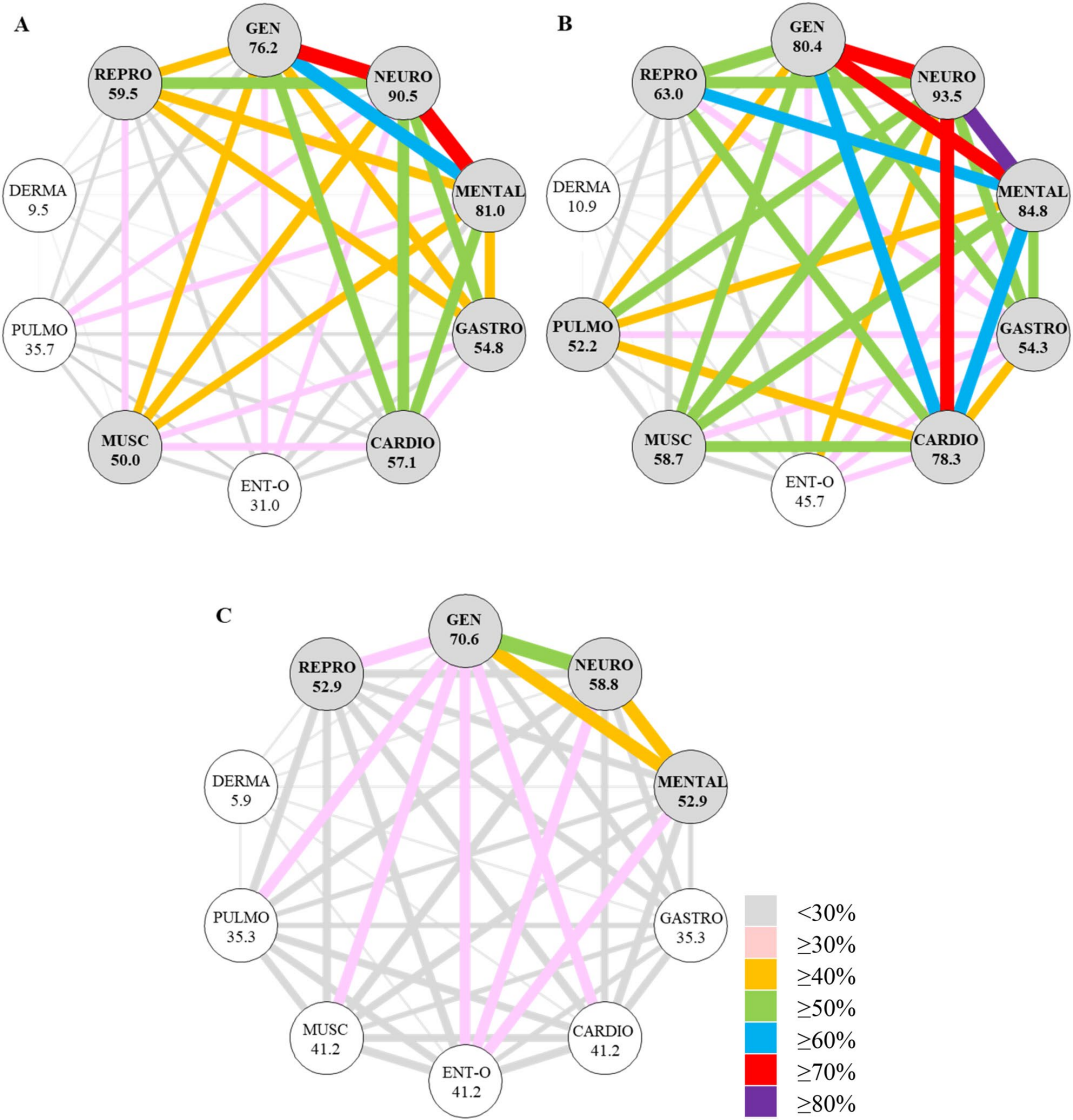
Networks of co-occurrence between symptoms in post COVID-19 condition according to the level of PA (**A**: low PA, **B**: moderate PA, **C**: high PA). The networks show a higher prevalence of symptoms and more prevalent co-occurrence between the symptoms in low and moderate PA groups compared to the high PA groups. *Notes* Organ systems, GEN: general, NEURO: neurological, GASTRO: gastrointestinal, CARDIO: cardiovascular, ENT-O: ear-nose-throat and ophthalmological, MUSC: musculoskeletal, PULMO: pulmonary, DERMA: dermatological, and REPRO: reproductive. PA: physical activity. The prevalence of symptoms in organ systems is shown in percentage.

The frequently occurring symptoms also showed a tendency to co-occur in PCC, especially in the low and moderate PA groups (Fig. 4).

Finally, 68.8% of the symptoms in PCC were intermittent and 31.2% were still present with a lower rate in the moderate PA group (28.5%; low PA: 34.9%, high PA: 34.7%).

The number of symptoms in acute COVID-19 and PCC showed a positive moderate to large correlation in the low (r(40) = 0.511, *p* = 0.001) and moderate (r(44) = 0.798, *p* < 0.001) PA groups. In the case of the high PA group, there was a non-significant association (r(17) = 0.451, *p* = 0.069).

### Functioning and changes in functioning based on the WHO Post COVID Case Report Form

**Figure 5:**
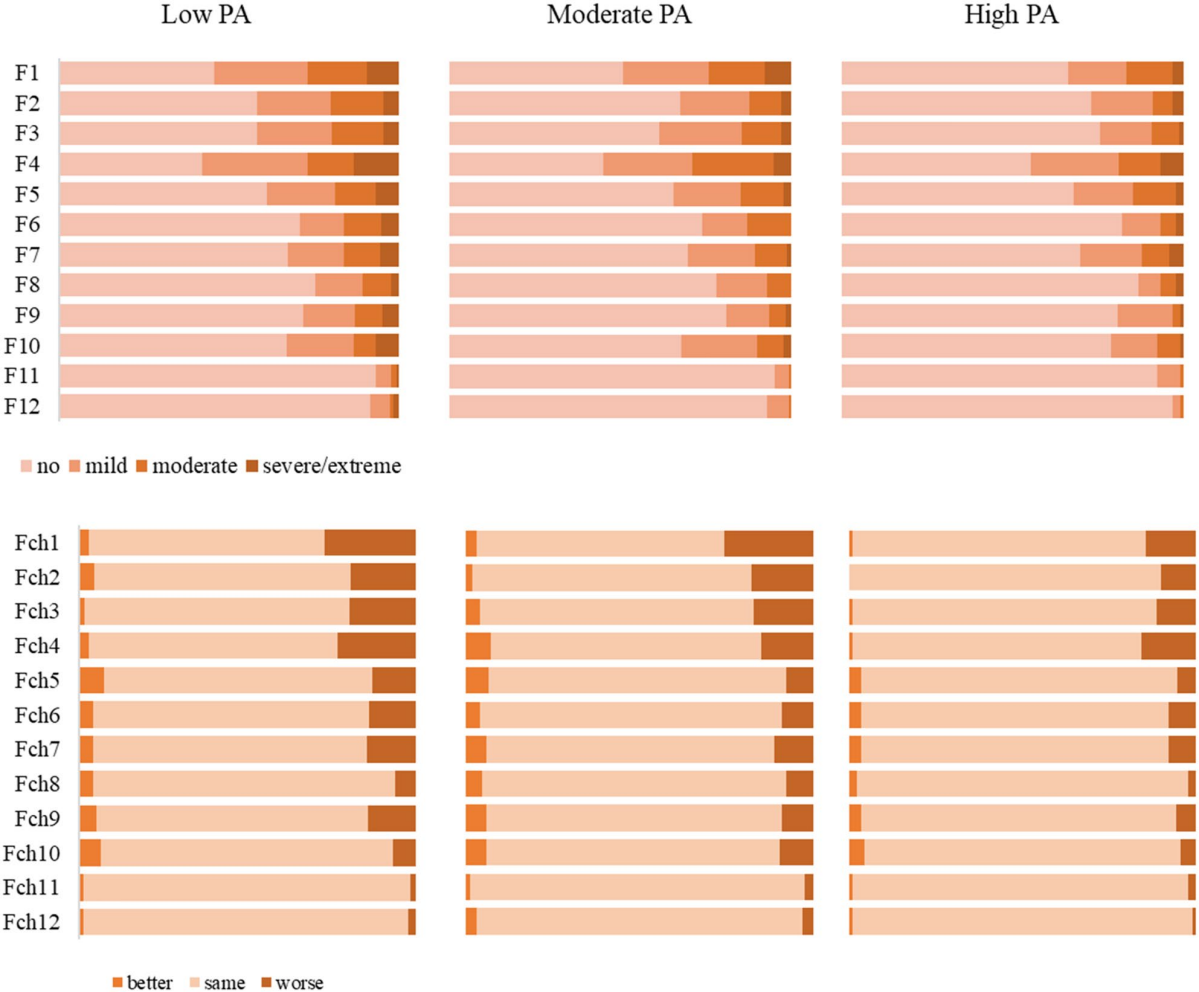
Functioning (F) and changes in functioning (Fch) among participants who contracted SARS-CoV-2 by the level of PA. *Notes* F/Fch1: being emotionally affected by health problems, F/Fch2: concentrating on doing something for 10 min, F/Fch3: standing for long periods, F/Fch4: day-to-day work/school, F/Fch5: dealing with people who do not know, F/Fch6: joining in community activities, F/Fch7: maintaining a friendship, F/Fch8: taking care of household responsibilities, F/Fch9: walking a long distance, F/Fch10: learning new task, F/Fch11: getting dressed, F/Fch12: washing whole body. PA: physical activity. The frequency (%) of the categories better, same, worse is represented in 100% stacked bar charts.

Among participants who had contracted SARS-CoV-2, the total score of functioning was between 0 and 72.5 (M = 11.78, SD = 13.22), which means mostly no/mild difficulties in functioning. The total score of changes in functioning compared to the period before COVID-19 was between 0 and 100 (M = 53.86, SD = 10.82), meaning that mostly no deterioration was reported. Overall, only five participants (four in the low PA group, one in the high PA group) experienced both at least moderate difficulties (functioning ≥ 50) and a worsened status in functioning since COVID-19 (changes in functioning > 50). The most frequently occurred difficult and/or worsened functions with a decreasing tendency according to the level of PA were: being emotionally affected by health problems (F/Fch1), concentrating on doing something for ten minutes (F/Fch2), standing for long periods (F/Fch3), day-to-day school/work ((F/Fch4) (Fig. 5).

## Discussion

This study aimed to examine the hypothesis that regular PA reduces the number of symptoms during and after COVID-19 infection in young women. We found that PCC is common among young women, in line with previous studies^37,38^. One-quarter of the women showed persisting, still present or intermittent—primarily neurological and mental—symptoms on average two years after infection.

It is well-known that regular PA can improve the immune system and can reduce the risk and severity of acute respiratory infections such as COVID-19 infection^39–41^. At the same time, the immune effect of PA depends on its FITT (frequency, intensity, time and type)^5,42^. Contrary to previous studies, we did not find a decreased prevalence of PCC according to the levels of PA among women^12,15,26,43^. At the same time, in line with the immune improving effect PA the number of symptoms was reduced both in acute COVID-19 and in PCC among young women, especially in the case of the high level of PA. Moreover, the rate of reinfection decreased with an increased level of PA.

Similarly to previous studies^12,13,44^, most young women also experienced neurological and mental symptoms in acute COVID-19 and PCC regardless of the level of PA. Previous studies found that physically active people had a lower number of symptoms in acute COVID-19 compared to inactive individuals^45,46^. Based on the results of the present study, the frequency of the most commonly reported symptoms in acute COVID-19 was lower in the high PA group compared to the low and moderate PA groups among young women.

In PCC, the symptoms were similar to the symptoms in acute COVID-19. At the same time, cardiovascular symptoms such as palpitations, chest pain, and post-exertional malaise were still more frequently present/intermittent in the moderate activity group. In addition, loss of interest/pleasure, anxiety, reduced smell and shortness of breath were also more commonly reported in the moderate PA group. Previous studies found that higher PA levels (in line with the global physical activity guidelines) were associated with less-severe and a smaller number of symptoms PCC^17,47^. In the present study, cardiovascular symptoms were more frequent in the moderate PA group regardless of the symptoms in acute COVID-19. In most cases, cardiovascular symptoms co-occurred with neurological symptoms.

Other studies showed that the intensity of regular physical activity is more crucial than other characteristics such as frequency, time, type or individual capacity^48^. However, it is controversial what the best intensity of physical activity is in different conditions^48,49^. Recent studies on people living with PCC found that fatigue and post-exertional malaise are frequently experienced^44,50^, although these symptoms may not only be caused by deconditioning^51,52^, and mental/neurological symptoms may also contribute to reduced exertional tolerance.

A significant decline was found in health-related quality of life during COVID-19 infection and in PCC but this decline is also related to age and the severity of COVID-19 infection^53,54^. After mild and moderate acute COVID-19, Lemhöfer et al.^55^ did not find a frequent or serious decrease in quality of life or work ability. In the present study, young women with PCC (and no other morbidity) showed mostly no/mild difficulties in functioning (quality of life), and there was no deterioration in the changes of functioning compared to the period before COVID-19. Mild difficulties in functioning are related to emotional and physical state, concentration and day-to-day school/work, which decreased according to the level of PA.

Physical activity is widely recognized as an important factor in maintaining a healthy life. Previous studies reported that the COVID-19 pandemic globally decreased physical activity^56,57^ which may be responsible for, at least in part, various physical and mental health conditions developing later. Indeed, people who remained physically active, involved in home-based exercises and fitness activities, were able to mitigate health-related problems of home confinement during and after the COVID-19 pandemic^58,59^. Improving skeletal muscle mass/function, exercise capacity, and cardiorespiratory fitness by high PA levels before contracting COVID-19 and soon after the acute COVID-19, likely helped to reduce the severity of PCC^20,21^ as also shown by the present study.

The complex pathogenesis of PCC has not yet been fully established since there are various persisting symptoms, for which the presence and interactions of several mechanisms can be responsible. In addition to physio-pathological mechanisms, psychological, mental, environmental, and social factors also play an important role in maintaining the symptoms after acute COVID-19^60,61^. Thus, it is important to elucidate these mechanisms in future studies. Among others, the early assessment of PCC, developing alternative medical and psychiatric diagnostic modalities, examining the role of confounding factors such as psychosocial characteristics of the people suffering from persisting symptoms and developing novel therapeutic methods for these complex pathological psychosomatic conditions.

## Limitations

The present study has some limitations. There is a possible selection bias as well as a recall bias since the study was conducted around two years after COVID-19 infection. It is important to highlight that it is difficult to distinguish the symptoms associated with long-term SARS-CoV-2 from the symptoms related to the pandemic. The study is a cross-sectional study; thus we did not have any information about the changes in the symptoms of PCC, which may continue, resolve or worsen. The potential confounders, and effect modifiers, such as psychosocial factors, which are also related to the level of PA, were not examined in the associations between PA and acute COVID-19 as well as PCC. Nevertheless, despite these limitations, the present study has many strengths: the homogeneous sample of young women with similar characteristics of COVID-19 infection, without comorbidities, and similar living situations excluded several biases of potential confounders.

## Conclusions

The salient finding of the present study is that a higher level of regular physical activity can reduce mental and neurological symptoms such as anxiety, depressed mood, fatigue, dizziness/light headedness, and forgetfulness both in acute COVID-19 and in PCC among young women. These symptoms may persist for a long time; thus, they should be followed up. In PCC, a higher level of regular physical activity can improve the quality of life not only by reducing the risk for the development of various mental and neurological symptoms but also by reducing the risk of reinfection, underlying the importance of regular PA, even in this and likely other viral disease conditions.

### Supplementary Information

Supplementary Information 1.

Supplementary Information 2.

## Data Availability

The datasets generated during and/or analysed during the current study are available from the corresponding author on reasonable request.
