# Supplementary material for: Higher level of physical activity reduces mental and neurological symptoms during and two years after COVID-19 infection in young women
Source: Sci Rep. 2024 Mar 22;14:6927. doi: 10.1038/s41598-024-57646-2 (PMC10960016; doi:10.1038/s41598-024-57646-2)
Supplement: Supplementary file 1 — Supplementary Information 1. [file 41598_2024_57646_MOESM1_ESM.docx]

Supplementary File 1. Frequency of symptoms in acute COVID-19 illness in the total sample and the activity categories.

| Symptoms in acute COVID-19 illness | Total sample | Activity categories | | | χ^2^ | *p* | V |
| --- | --- | --- | --- | --- | --- | --- | --- |
|  |  | low | moderate | high |  |  |  |
|  | %(n) | %(n) | %(n) | %(n) |  |  |  |
| fever | 24.4(104) | 28.1(48) | 21.9(37) | 22.1(19) | 2.072 | 0.355 | 0.07 |
| light fever | 33.7(140) | 37.3(63) | 32.1(53) | 29.3(24) | 1.874 | 0.392 | 0.07 |
| loss of appetite | 36.2(154) | 41.9(72) | 35.1(59) | 26.7(23) | 5.804 | 0.055 | 0.12 |
| **persistent fatigue** | 58.7(250) | **65.1(112)** | **57.4(97)** | **48.2(41)** | **6.878** | **0.032** | **0.13** |
| weight loss | 17.8(75) | 18.8(32) | 18.8(31) | 14.0(12) | 1.101 | 0.577 | 0.05 |
| cannot move or feel one side of body/face | 5.2(22) | 7.6(13) | 3.6(6) | 3.6(3) | 3.394 | 0.183 | 0.09 |
| dizziness/light headedness | 51.9(221) | 54.7(94) | 54.2(91) | 41.9(36) | 4.339 | 0.114 | 0.10 |
| fainting/blackouts | 13.1(56) | 14.5(25) | 12.4(21) | 11.6(10) | 0.542 | 0.763 | 0.04 |
| **forgetfulness** | 43.2(183) | **48.3(83)** | **43.7(73)** | **31.8(27)** | **6.341** | **0.042** | **0.12** |
| numbness or tingling | 20.9(89) | 21.6(37) | 22.6(38) | 16.3(14) | 1.465 | 0.481 | 0.06 |
| persistent headache | 30.1(128) | 35.1(60) | 27.8(47) | 24.7(21) | 3.617 | 0.164 | 0.09 |
| problems passing urine | 7.6(32) | 8.2(14) | 7.8(13) | 5.8(5) | 0.498 | 0.780 | 0.03 |
| problems with balance | 13.9(59) | 18.1(31) | 13.0(22) | 7.1(6) | 5.864 | 0.053 | 0.12 |
| seizures | 15.6(66) | 17.6(30) | 13.8(23) | 15.3(13) | 0.968 | 0.616 | 0.05 |
| slowness of movement | 14.3(61) | 19.2(33) | 11.3(19) | 10.5(9) | 5.602 | 0.061 | 0.12 |
| sleeping less | 25.2(107) | 26.0(44) | 24.3(41) | 25.6(22) | 0.148 | 0.929 | 0.02 |
| **sleeping more** | 33.3(141) | **40.9(70)** | **31.1(52)** | **22.1(19)** | **9.709** | **0.008** | **0.15** |
| tremors | 18.3(77) | 17.8(30) | 20.8(35) | 14.3(12) | 1.661 | 0.436 | 0.06 |
| **trouble in concentrating** | 43.5(184) | **50.3(86)** | **43.1(72)** | **30.6(26)** | **8.986** | **0.011** | **0.15** |
| **weakness in limbs** | 18.2(77) | **24.6(42)** | **15.5(26)** | **10.6(9)** | **8.808** | **0.012** | **0.14** |
| **anxiety** | 56.1(239) | **55.0(94)** | **63.9(108)** | **43.0(37)** | **10.241** | **0.006** | **0.16** |
| behaviour change | 27.8(116) | 29.8(50) | 28.5(47) | 22.4(19) | 1.618 | 0.445 | 0.06 |
| **depressed mood** | 53.3(227) | **56.7(97)** | **56.8(96)** | **39.5(34)** | **8.186** | **0.017** | **0.14** |
| **loss of interest/pleasure** | 52.6(224) | **59.3(102)** | **53.6(90)** | **37.2(32)** | **11.333** | **0.003** | **0.16** |
| hallucinations | 9.1(39) | 10.5(18) | 7.1(12) | 10.5(9) | 1.393 | 0.498 | 0.06 |
| constipation | 14.9(63) | 15.8(27) | 16.3(27) | 10.5(9) | 1.685 | 0.431 | 0.06 |
| diarrhoea | 25.4(108) | 25.1(43) | 27.4(46) | 22.1(19) | 0.850 | 0.654 | 0.05 |
| nausea/vomiting | 20.5(87) | 22.7(39) | 19.6(33) | 17.6(15) | 1.000 | 0.607 | 0.05 |
| problem swallowing | 8.9(38) | 11.1(19) | 8.3(14) | 5.8(5) | 2.116 | 0.347 | 0.07 |
| stomach pain | 20.4(87) | 22.8(39) | 21.3(36) | 14.0(12) | 2.893 | 0.235 | 0.08 |
| chest pain | 34(144) | 33.1(57) | 35.9(60) | 31.8(27) | 0.523 | 0.770 | 0.04 |
| palpitations | 37.1(157) | 38.5(65) | 39.6(67) | 29.4(25) | 2.756 | 0.252 | 0.08 |
| post-exertional malaise | 25.9(110) | 29.7(51) | 25.7(43) | 18.6(16) | 3.650 | 0.161 | 0.09 |
| problems hearing | 16.0(68) | 19.3(33) | 14.2(24) | 12.8(11) | 2.454 | 0.293 | 0.08 |
| problems seeing | 13.4(57) | 12.9(22) | 14.3(24) | 12.8(11) | 0.171 | 0.918 | 0.02 |
| reduced smell | 32.4(138) | 37.2(64) | 27.2(46) | 32.9(28) | 3.899 | 0.142 | 0.10 |
| reduced taste | 29.8(127) | 30.4(52) | 26.6(45) | 34.9(30) | 1.906 | 0.386 | 0.07 |
| ringing in ears | 15.1(64) | 18.3(31) | 14.2(24) | 10.5(9) | 2.935 | 0.230 | 0.08 |
| jerking of limbs | 18.1(76) | 20.1(34) | 16.1(27) | 17.9(15) | 0.935 | 0.626 | 0.05 |
| joint pain/swelling | 27.8(118) | 30.8(53) | 25.7(43) | 25.9(22) | 1.283 | 0.526 | 0.06 |
| pain on breathing | 24.3(103) | 26.3(45) | 25.4(43) | 17.9(15) | 2.394 | 0.302 | 0.08 |
| persistent muscle pain | 16.7(71) | 19.8(34) | 14.3(24) | 15.1(13) | 2.025 | 0.363 | 0.07 |
| problems with gait/falls | 9.2(39) | 11.6(20) | 8.9(15) | 4.7(4) | 3.304 | 0.192 | 0.09 |
| stiffness of muscles | 8.5(36) | 10.5(18) | 7.1(12) | 7.0(6) | 1.515 | 0.469 | 0.06 |
| swollen ankles | 5.6(24) | 7.6(13) | 4.1(7) | 4.8(4) | 2.021 | 0.364 | 0.07 |
| persistent dry cough | 27.7(118) | 30.2(52) | 28.0(47) | 22.1(19) | 1.907 | 0.385 | 0.07 |
| shortness of breath | 31.4(134) | 35.5(61) | 27.8(47) | 30.2(26) | 2.385 | 0.303 | 0.08 |
| lumpy lesions | 4.9(21) | 7.6(13) | 1.8(3) | 5.9(5) | 6.229 | 0.044 | 0.12 |
| skin rash | 8.5(36) | 10.5(18) | 8.3(14) | 4.7(4) | 2.440 | 0.295 | 0.08 |
| dysmenorrhea | 54.7(233) | 57.6(99) | 55.4(93) | 47.7(41) | 2.309 | 0.315 | 0.07 |
