# Supplementary material for: Higher level of physical activity reduces mental and neurological symptoms during and two years after COVID-19 infection in young women
Source: Sci Rep. 2024 Mar 22;14:6927. doi: 10.1038/s41598-024-57646-2 (PMC10960016; doi:10.1038/s41598-024-57646-2)
Supplement: Supplementary file 2 — Supplementary Information 2. [file 41598_2024_57646_MOESM2_ESM.docx]

Supplementary File 2. Frequency of symptoms in post COVID-19 condition in the total sample and in the activity categories.

| Symptoms in post COVID-19 condition | Total sample | Activity categories | | | χ^2^ | *p* | V |
| --- | --- | --- | --- | --- | --- | --- | --- |
|  |  | low | moderate | high |  |  |  |
|  | %(n) | %(n) | %(n) | %(n) |  |  |  |
| fever | 5.3(6) | 6.5(3) | 4.3(2) | 4.8(1) | –* | – | – |
| light fever | 16.7(19) | 17.4(8) | 21.3(10) | 4.8(1) | – | – | – |
| loss of appetite | 35.1(40) | 32.6(15) | 42.6(20) | 23.8(5) | 2.447 | 0.294 | 0.15 |
| persistent fatigue | 63.2(72) | 56.5(26) | 74.5(35) | 52.4(11) | 4.503 | 0.105 | 0.20 |
| weight loss | 12.3(14) | 8.7(4) | 14.9(7) | 14.3(3) | – | – | – |
| cannot move or feel one side of body/face | 1.8(2) | 4.3(2) | 0(0 | 0(0) | – | – | – |
| **dizziness/light headedness** | 51.3(59) | **52.2(24)** | **64.6(31)** | **19.0(4)** | **12.148** | **0.002** | **0.33** |
| fainting/blackouts | 12.3(14) | 13.0(6) | 12.8(6) | 9.5(2) | – | – | – |
| **forgetfulness** | 46.1(53) | **45.7(21)** | **56.2(27)** | **23.8(5)** | **6.193** | **0.045** | **0.23** |
| numbness or tingling | 23.7(27) | 15.2(7) | 31.9(15) | 23.8(5) | – | – | – |
| persistent headache | 27.0(31) | 23.9(11) | 31.2(15) | 23.8(6) | 0.771 | 0.680 | 0.08 |
| problems passing urine | 3.5(4) | 4.3(2) | 4.3(2) | 0(0) | – | – | – |
| problems with balance | 10.5(12) | 15.2(7) | 10.6(5) | 0(0) | – | – | – |
| seizures | 14.0(16) | 6.5(3) | 21.3(10) | 14.3(3) | – | – | – |
| slowness of movement | 7.9(9) | 8.7(4) | 8.5(4) | 4.8(1) | – | – | – |
| sleeping less | 21.1(24) | 23.9(11) | 19.1(9) | 19.0(4) | – | – | – |
| sleeping more | 28.9(33) | 28.3(13) | 36.2(17) | 14.3(3) | 3.397 | 0.183 | 0.17 |
| tremors | 13.2(15) | 8.7(4) | 21.3(10) | 4.8(1) | – | – | – |
| trouble in concentrating | 40.9(47) | 39.1(18) | 50.0(24) | 23.8(5) | 4.243 | 0.120 | 0.19 |
| weakness in limbs | 12.3(14) | 15.2(7) | 14.9(7) | 0(0) | – | – | – |
| **anxiety** | 43.9(50) | **39.1(18)** | **66.0(31)** | **4.8(1)** | **22.776** | **<0,001** | **0.45** |
| behaviour change | 22.8(26) | 19.6(9) | 29.8(14) | 14.3(3) | – | – | – |
| **depressed mood** | 43.0(49) | **43.5(20)** | **55.3(26** | **14.3(3)** | **9.980** | **0.007** | **0.30** |
| **loss of interest/pleasure** | 52.6(60) | **43.5(20)** | **68.1(32)** | **38.1(8)** | **7.828** | **0.020** | **0.26** |
| hallucinations | 4.4(5) | 4.3(2) | 6.4(3) | 0(0) | – | – | – |
| constipation | 15.7(18) | 15.2(7) | 18.8(9) | 9.5(2) | – | – | – |
| diarrhoea | 25.4(29) | 30.4(14) | 23.4(11) | 19.0(4) | 1.160 | 0.560 | 0.10 |
| nausea/vomiting | 16.7(19) | 13.0(6) | 23.4(11) | 9.5(2) | – | – | – |
| problem swallowing | 5.3(6) | 4.3(2) | 8.5(4) | 0(0) | – | – | – |
| stomach pain | 27.2(31) | 28.3(13) | 29.8(14) | 19.0(4) | 0.890 | 0.641 | 0.09 |
| ***chest pain*** | 30.4(35) | ***26.1(12)*** | ***41.7(20)*** | ***14.3(3)*** | ***5.858*** | ***0.053*** | ***0.23*** |
| **palpitations** | 42.1(48) | **37.0(17)** | **57.4(27)** | **19.0(4)** | **9.618** | **0.008** | **0.29** |
| **post-exertional malaise** | 25.4(29) | **17.4(8)** | **38.3(18)** | **14.3(3)** | **7.045** | **0.030** | **0.25** |
| problems hearing | 16.7(19) | 19.6(9) | 17.0(8) | 9.5(2) | – | – | – |
| problems seeing | 9.6(11) | 6.5(3) | 14.9(7) | 4.8(1) | – | – | – |
| **reduced smell** | 24.3(28) | ***17.4(8)*** | ***35.4(17)*** | ***14.3(3)*** | ***5.556*** | ***0.062*** | ***0.22*** |
| reduced taste | 13.2(15) | 8.7(4) | 14.9(7) | 19.0(4) | – | – | – |
| ringing in ears | 12.3(14) | 8.7(4) | 17.0(8) | 9.5(2) | – | – | – |
| jerking of limbs | 16.7(19) | 17.4(8) | 19.1(9) | 9.5(2) | – | – | – |
| joint pain/swelling | 31.6(36) | 30.4(14) | 36.2(17) | 23.8(5) | 1.073 | 0.585 | 0.10 |
| pain on breathing | 20.9(24) | 15.2(7) | 29.2(14) | 14.3(3) | – | – | – |
| persistent muscle pain | 13.2(15) | 8.7(4) | 17.0(8) | 14.3(3) | – | – | – |
| problems with gait/falls | 7.9(9) | 10.9(5) | 8.5(4) | 0(0) | – | – | – |
| stiffness of muscles | 7.9(9) | 8.7(4) | 8.5(4) | 4.8(1) | – | – | – |
| swollen ankles | 4.4(5) | 6.5(3) | 4.3(2) | 0(0) | – | – | – |
| persistent dry cough | 21.1(24) | 17.4(8) | 27.7(13) | 14.3(3) | – | – | – |
| **shortness of breath** | 26.3(30) | **19.6(9)** | **38.3(18)** | **14.3(3)** | **6.128** | **0.047** | **0.230** |
| lumpy lesions | 3.5(4) | 4.3(2) | 4.3(2) | 0(0) | – | – | – |
| skin rash | 5.3(6) | 4.3(2) | 6.4(3) | 4.8(1) | – | – | – |
| dysmenorrhea | 55.3(63) | 54.3(25) | 61.7(29) | 42.9(8) | 2.111 | 0.348 | 0.140 |

*Notes.* * the expected count of cells was less than 5.
